# Central Venous Disease Increases the Risk of Microbial Colonization in Hemodialysis Catheters

**DOI:** 10.3389/fmed.2021.645539

**Published:** 2021-08-23

**Authors:** Xianhui Liang, Yamin Liu, Bohan Chen, Ping Li, Peixiang Zhao, Zhangsuo Liu, Pei Wang

**Affiliations:** ^1^Blood Purification Center, Institute of Nephrology, the First Affiliated Hospital of Zhengzhou University, Zhengzhou, China; ^2^Research Institute of Nephrology, Zhengzhou University, Zhengzhou, China

**Keywords:** tunneled cuffed catheter, catheter-related infection, central venous stenosis, central venous thrombosis, microbial colonization

## Abstract

**Objectives:** Tunneled-cuffed catheters (TCCs) are widely used in maintenance hemodialysis patients. However, microbial colonization in catheters increases the likelihood of developing various complications, such as catheter-related infection (CRI), catheter failure, hospitalization, and death. Identification of the risk factors related to microorganism colonization may help us reduce the incidence of these adverse events. Therefore, a retrospective analysis of patients who underwent TCC removal was conducted.

**Methods:** From a pool of 389 adult patients, 145 were selected for inclusion in the study. None of the patients met the diagnostic criteria for CRI within 30 days before recruitment. The right internal jugular vein was the unique route evaluated. The catheter removal procedure was guided by digital subtraction angiography. Catheter tips were collected for culture. Biochemical and clinical parameters were collected at the time of catheter removal.

**Results:** The average age of this cohort was 55.46 ± 17.25 years. A total of 45/145 (31.03%) patients were verified to have a positive catheter culture. The proportions of gram-positive bacteria, gram-negative bacteria, and fungi were 57.8, 28.9, and 13.3%, respectively. History of CRI [odds ratio (OR) = 2.44, 95% confidence interval (CI) 1.09 to 5.49], fibrin sheath (OR = 2.93, 95% CI 1.39–6.19), white blood cell (WBC) count ≥5.9 × 10^9^/l (OR = 2.31, 95% CI 1.12–4.77), moderate (OR = 4.87, 95% CI 1.61–14.78) or severe central venous stenosis (CVS) (OR = 4.74, 95% CI 1.16–19.38), and central venous thrombosis (CVT) (OR = 3.41, 95% CI 1.51–7.69) were associated with a significantly increased incidence of microbial colonization in a univariate analysis. Central venous disease (CVD) elevated the risk of microbial colonization, with an OR of 3.37 (1.47–7.71, *P* = 0.004). A multivariate analysis showed that both CVS and CVT were strongly associated with catheter microbial colonization, with ORs of 3.06 (1.20–7.78, *P* = 0.019) and 4.13 (1.21–14.05, *P* = 0.023), respectively. As the extent of stenosis increased, the relative risk of catheter microbial colonization also increased. In patients with moderate and severe stenosis, a sustained and significant increase in OR from 5.13 to 5.77 was observed.

**Conclusions:** An elevated WBC count and CVD can put hemodialysis patients with TCCs at a higher risk of microbial colonization, even if these patients do not have the relevant symptoms of infection. Avoiding indwelling catheters is still the primary method for preventing CRI.

## Introduction

Hemodialysis remains the main component of renal replacement therapy (RRT) worldwide. Well-functioning vascular access, a fundamental aspect of hemodialysis, has always been an area of great concern (1–3). Arteriovenous access, including arteriovenous fistulas (AVFs) and arteriovenous grafts (AVGs), is often characterized by longer patency and fewer complications and is also considered to be more ideal than central venous catheters (CVCs) (4, 5). However, a large proportion of patients (~45%) maintain their treatment through catheters based on the following considerations: late referral for dialysis, critical illness, poor peripheral vascular conditions, or low acceptance of arteriovenous access (6). Indwelling catheters are believed to be the primary source of systemic infection, aptly referred to as catheter-related infections (CRIs); therefore, indwelling catheters put patients at increased risks for catheter failure, death, hospitalization, and other adverse events (7–9). Hence, efforts to limit the use of CVCs and lessen the incidence of CRIs have become a focus of research (10, 11). Increasing evidence indicates that microbial colonization of a catheter is closely related to but often precedes bloodstream infection in hemodialysis patients (12–15). The risk of developing CRIs is dramatically increased in patients with positive catheter cultures (16). Strategies intended to prevent or eliminate microorganism colonization may be able to provide a substantial breakthrough in the care of patients with catheters (17). In this retrospective study, we collected the clinical data of patients with positive catheter cultures who had no evidence of a catheter-related blood stream infection (CRBSI); we also examined the factors involved in microbial colonization and central venous disease (CVD).

## Materials and Methods

### Study Design

**Figure 1:**
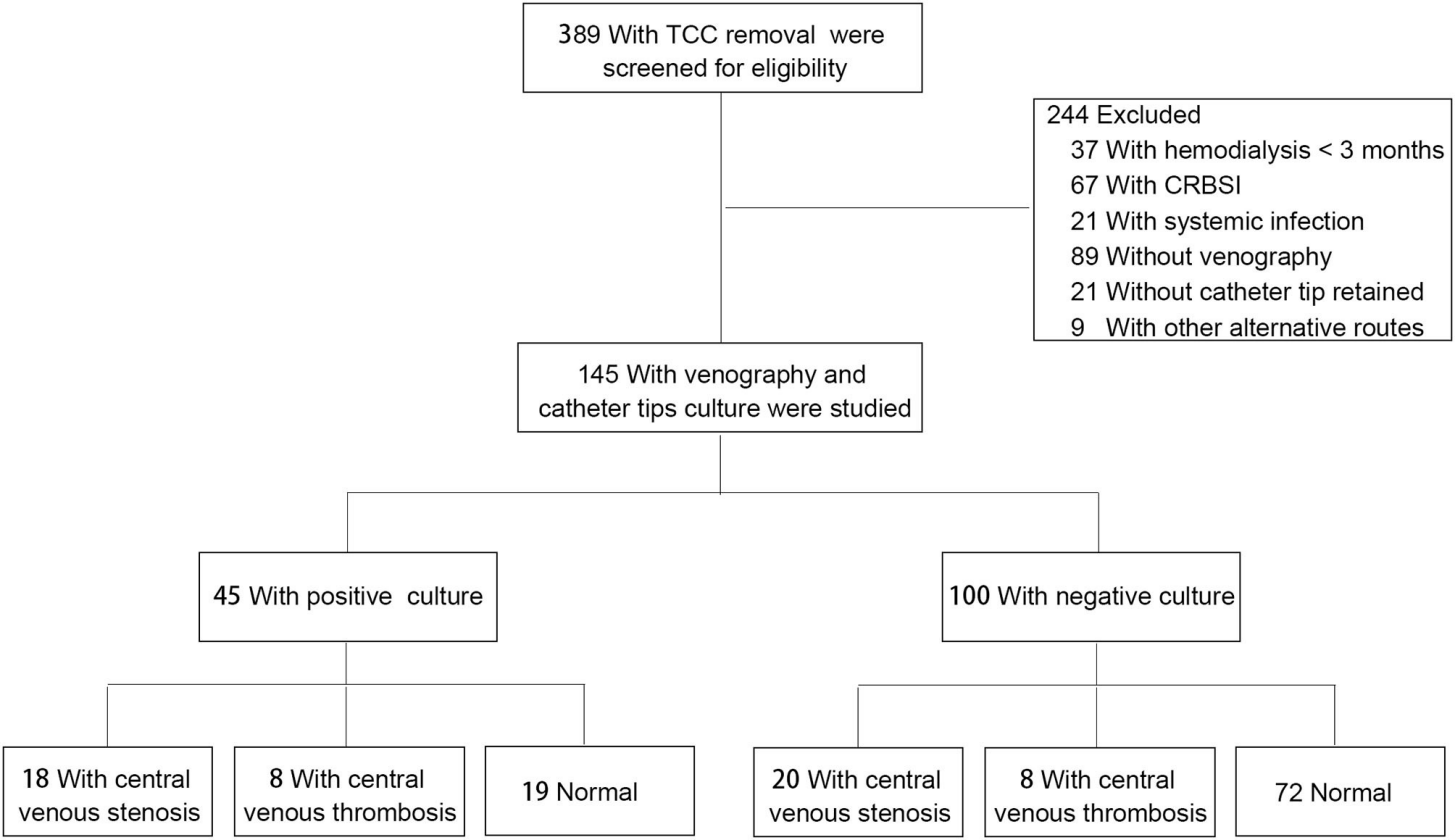
Flow diagram depicting the design of the retrospective cohort study.

For this retrospective cohort study, a total of 389 adult patients who underwent tunneled-cuffed catheter (TCC) removal in our center between June 2019 and June 2020 were screened for inclusion (Figure 1). None of the patients met the diagnostic criteria for CRIs within 30 days before recruitment. The right internal jugular vein was the unique route chosen for analysis; patients with access through other alternative routes were excluded. Patients with incomplete clinical data, systemic infection, and exit-site or tunnel infection were not included. The procedure was guided by digital subtraction angiography and performed with strict aseptic techniques under local anesthesia. Antimicrobial lock solutions are not routinely used to prevent CRBSI; however, we usually use a 1,500-U/ml heparin sodium lock solution, and urokinase is used every 2–4 weeks to prevent catheter-associated thrombus. When anticoagulation was contraindicated, the patients received sodium chloride (10%). Finally, 145 patients were enrolled in this study for further assessment. Demographic data as well as clinical parameters were documented, including sex, age, primary cause of end-stage kidney disease, history of CRI, catheter indwelling time, presence of CVD, hemoglobin, platelet count, etc.

Catheter removal was performed when the catheter was no longer needed or the catheter was no longer able to be used. Successful kidney transplantation, maturation of arteriovenous access, external catheter breakage, catheter loosening or even falling out, and catheter dysfunction were common reasons for removal. After TCC removal, the follow process was performed: (1) A 5-cm segment of each catheter tip was collected and cultured. (2) Patients with an AVF or AVG that was ready for use were changed to arteriovenous access. (3) New peripheral vascular access was established for patients who preferred AVF or AVG. Usually, a new TCC was implanted through the femoral vein to maintain dialysis treatment before a patient was converted to arteriovenous access. Early cannulation grafts were used in patients when suitable. (4) For patients who still needed a catheter, a new TCC could be inserted over a guidewire during *in situ* exchange or after repuncture. Biochemical and clinical parameters were collected at the time of catheter removal. Catheter tip cultures were performed by semi-quantitative culture on blood agar plates in the Microbiology Laboratory of the First Affiliated Hospital of Zhengzhou University. The plate was incubated in a 35°C incubator for 48 h. The catheter tip cultures were considered positive if 15 or more colony-forming units (CFUs) were observed.

CVD was defined as central venous stenosis (CVS) or central venous thrombosis (CVT), both of which were diagnosed with digital subtraction angiography by two or more experienced vascular access physicians. CVT was defined as a filling defect in the lumen of the vessel, and CVS was defined as a narrow lumen. Stenosis was classified as mild, moderate, or severe according to the degree of stenosis: <50, 50–70, or ≥70%, respectively.

### Statistical Analysis

Continuous variables are summarized using the mean ± SD or median (interquartile range). Categorical variables are summarized using numbers and percentages; continuous variables were analyzed by using *t*-tests and Mann–Whitney *U* tests, as appropriate. Categorical variables were compared using the χ^2^ test or Fisher's exact test. Logistic binary regression was applied to examine the association between catheter microbial colonization and the resulting variables. *P* < 0.05 was considered statistically significant.

## Results

### Patient Characteristics

The average age of this cohort was 55.46 ± 17.25 years. The male-to-female ratio was 1.46:1. Diabetes mellitus, chronic glomerulonephritis, and hypertension were the top three reasons for RRT. Positive catheter cultures were detected in a total of 45/145 (31.03%) patients, whereas 100/145 (68.97%) control patients had negative cultures. The mean indwelling catheter times for patients with and without microbial colonization were 25.6 ± 20.30 months and 26.91 ± 24.61 months, respectively. The patient characteristics (Table 1) revealed that, compared with the negative group, the positive group had a higher prevalence of CVD (57.8 vs. 28%, *P* = 0.001), more CVT (17.8 vs. 8.0%), more serious CVS (moderate stenosis, 24.3 vs. 7.6%; serious stenosis 13.5 vs. 4.3%), and a higher percentage of fibrin sheaths (46.7 vs. 23%, *P* = 0.004). Previous CRIs, a white blood cell (WBC) count over 5.9 × 10^9^/l, and elevated C-reactive protein also increased the risk for microbial colonization.

**Table 1:** Clinical and laboratory data of patients in this retrospective cohort study.

**Variable**	**Positive group** **(*N* = 45, %)**	**Negative group** **(*N* = 100, %)**	***P***	**OR (95%CI)**	***P***
Sex			0.632		0.632
Male	28 (62.2)	58 (58)		1	
Female	17 (37.8)	42 (42)		0.84 (0.41–1.73)	
Age			0.537		0.537
<60	25 (55.6)	61 (61.0)		1	
≥60	20 (44.4)	39 (39.0)		1.25 (0.61–2.55)	
Primary disease			0.277		0.288
Others	3 (6.7)	8 (8)		1	
Diabetic nephropathy	22 (48.8)	30 (30.0)		1.96 (0.46–8.22)	0.360
Hypertension	7 (15.6)	18 (18)		1.04 (0.21–5.08)	0.964
Chronic glomerulonephritis	10 (22.2)	35 (35)		0.76 (0.17–3.42)	0.723
Polycystic kidney	3 (6.7)	9 (9)		0.89 (0.14–5.72)	0.901
Diabetes mellitus	22 (48.9)	39 (39.0)	0.264	1.50 (0.74–3.04)	0.266
History of CRI	15 (33.3)	17 (17.0)	0.028	2.44 (1.09–5.49)	0.031
Reason for catheter removal			0.067	1	0.075
AVF, AVG maturation	9 (20)	34 (34.0)			
Kidney transplantation	4 (8.9)	18 (18.0)		0.84 (0.23–3.11)	0.793
Mechanical complication	4 (8.9)	4 (4.0)		3.78 (0.79–18.13)	0.097
Catheter dysfunction	28 (62.2)	44 (44.0)		2.40 (1.0–5.76)	0.049
Catheter indwelling time			0.276		0.277
<20 months	19 (42.2)	52 (52.0)		1	
≥20 months	26 (57.8)	48 (48.0)		1.48 (0.73–3.01)	
Central venous disease	26 (57.8)	28 (28.0)	0.001	3.52 (1.69–7.34)	0.001
**Type of disease**			0.003		0.004
Normal	19 (42.2)	72 (72.0)		1	
CVT	8 (17.8)	8 (8.0)		3.41 (1.51–7.69)	0.003
CVS	18 (40)	20 (20.0)		3.79 (1.26–11.41)	0.018
**Degree of stenosis**^***#***^			0.007		0.013
Normal	19 (51.4)	72 (78.3)		1	
Mild	4 (10.8)	9 (9.8)		1.68 (1.47–6.07)	0.425
Moderate	9 (24.3)	7 (7.6)		4.87 (1.61–14.78)	0.005
Severe	5 (13.5)	4 (4.3)		4.74 (1.16–19.38)	0.030
Fibrin sheath	21 (46.7)	23 (23.0)	0.004	2.93 (1.39–6.19)	0.005
WBC count (10^9^/l)			0.023		0.024
<5.9	16 (35.6)	56 (56.0)		1	
≥5.9	29 (65.4)	44 (44.0)		2.31 (1.12–4.77)	
Hemoglobin (g/l)	103.87 ± 19.28	105.64 ± 18.78	0.604	1.00 (0.98–1.01)	0.601
Serum albumin (g/l)	38.87 ± 4.09	40.32 ± 6.13	0.097	0.95 (0.90–1.02)	0.153
Serum ferritin (ng/ml)	275.70 (179.02, 465.80)	246.40 (109.88, 418.70)	0.054	1.00	0.852
C-reactive protein (μg/ml)	13.10 (7.05, 20.49)	5.43 (2.46, 12.42)	<0.001	1.02 (1.00–1.05)	0.079

# *CVS is graded according to the degree of stenosis, <50%, 50%−70%, ≥70% are mild, moderate, and severe stenosis, respectively. The catheter indwelling time and WBC count are divided into two groups according to the median. Data are expressed as mean ± SD, median (interquartile range), or number*.

### Microbiological Characteristics

Among the 45 positive cultures, the proportion due to gram-positive bacteria was comparatively higher, and the proportions of gram-positive bacteria, gram-negative bacteria, and fungi were 57.8, 28.9, and 13.3% (Table 2), respectively. *Staphylococcus epidermidis* was the overwhelming primary pathogen, accounting for 65.4% of gram-positive bacteria, followed by *Pseudomonas aeruginosa, Escherichia coli*, and *Staphylococcus aureus*. All six strains of fungi were *Candida parapsilosis*.

**Table 2:** Distribution of positive pathogens.

**Pathogens**	**Numbers**	**Proportion (%)**
**Gram-positive bacteria**	26	57.8
*Staphylococcus epidermidis*	17	
*Staphylococcus aureus*	2	
*Brevibacterium casei*	1	
*Staphylococcus hominis*	1	
*Staphylococcus haemolyticus*	1	
*Staphylococcus capitis*	1	
*Corynebacterium striata*	1	
*Staphylococcus wokerii*	1	
*Streptococcus angina*	1	
**Gram-negative bacteria**	13	28.9
*Pseudomonas aeruginosa*	3	
*Escherichia coli*	2	
*Actinobacillus rhizobium*	1	
*Acinetobacter lophi*	1	
*Achromobacter xylosoxidans*	1	
*Proteus mirabilis*	1	
*Stenotrophomonas maltophilia*	1	
*Enterobacter cloacae complex*	1	
*Enterobacter cloacae subsp. Cloacae*	1	
*Klebsiella oxytoca*	1	
**Fungus**	6	
*Candida parapsilosis*	6	13.3

### Analysis of Risk Factors for Microbial Colonization

As shown in Table 1, the number of patients with a history of CRI, fibrin sheath, elevated WBC count, and moderate to severe CVS and CVT was substantially different between the positive and negative groups. To determine whether these variables gave rise to microbial colonization of the catheter, we further conducted univariate logistic regression analyses. The results are shown in the right column of Table 1. History of CRI [odds ratio (OR) = 2.44, 95% confidence interval (CI) 1.09–5.49], fibrin sheath (OR = 2.93, 95% CI 1.39–6.19), WBC count ≥5.9 × 10^9^/l (OR = 2.31, 95% CI 1.12–4.77), moderate CVS (OR = 4.87, 95% CI 1.61–14.78) or severe CVS (OR = 4.74, 95% CI 1.16–19.38), and CVT (OR = 3.41, 95% CI 1.51–7.69) were associated with a significantly increased incidence of microbial colonization.

**Figure 2:**
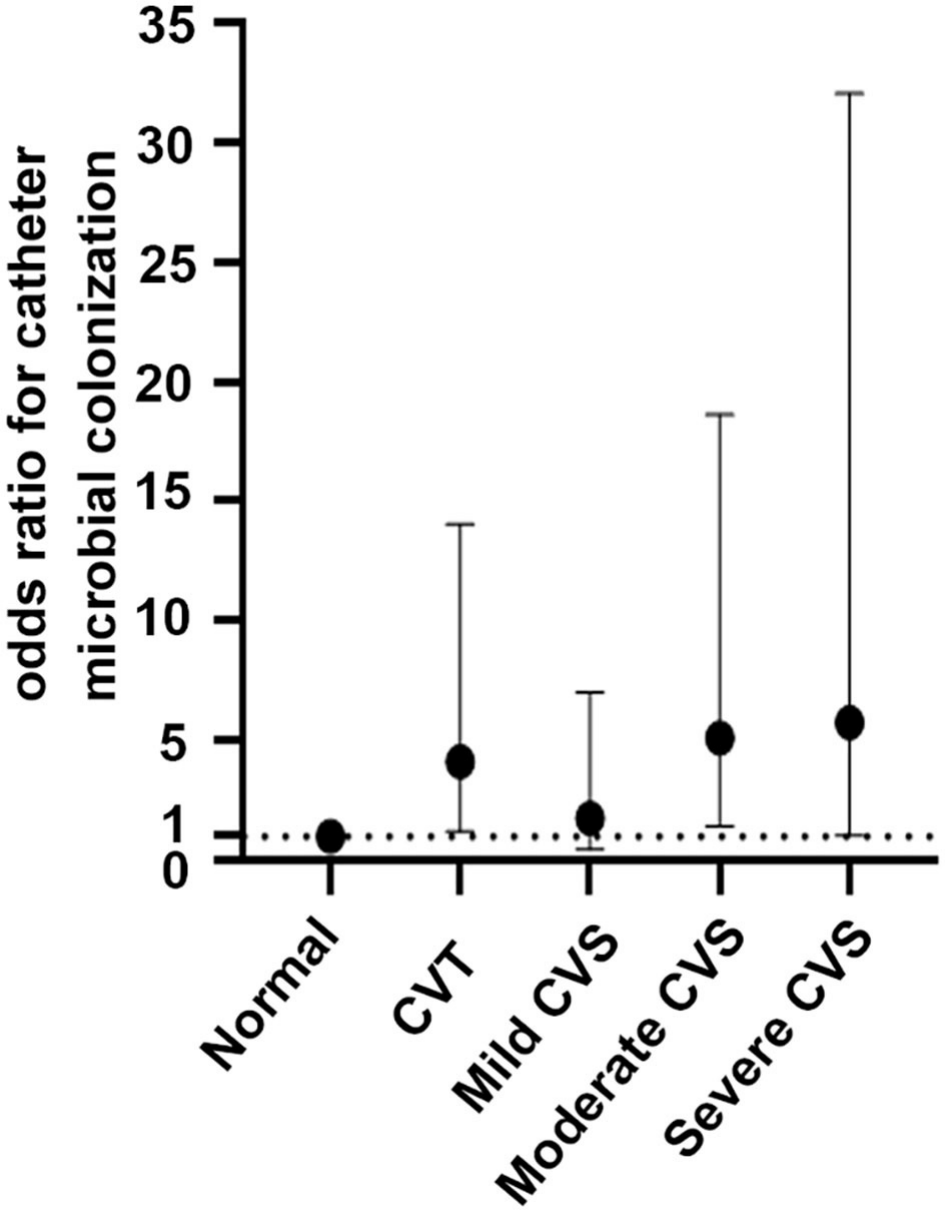
Odds ratios for catheter microbial colonization across the central venous diseases. A dashed reference line for normal is drawn at OR, 1. Mild CVS: OR, 1.76; 95% CI, 0.44–7.02; *P* = 0.425; moderate CVS: OR, 5.13; 95% CI, 1.41–18.63; *P* = 0.013; severe CVS: OR, 5.77; 95% CI, 1.04–32.04; *P* = 0.045; CVT: OR, 4.13; 95% CI, 1.21–14.05, *P* = 0.023.

Multivariate logistic regression (Table 3, Figure 2) was adjusted for age, sex, presence of diabetes mellitus, catheter indwelling time, fibrin sheath, history of CRI, and elevated WBC count and indicated that CVD elevated the risk of microbial colonization with an OR of 3.37 (1.47–7.71, *P* = 0.004). In model 2, when CVD was classified as thrombus or stenosis, the results suggested that both CVT and CVS were strongly associated with catheter microbial colonization, with ORs of 3.06 (1.20–7.78, *P* = 0.019) and 4.13 (1.21–14.05, *P* = 0.023), respectively. In model 3, CVS was further divided into mild, moderate, and severe stenosis. As the extent of stenosis increased, the relative risk of catheter microbial colonization also increased. In patients with moderate and severe stenosis, a sustained and significant increase in risk was found, with an OR ranging from 5.13 to 5.77.

**Table 3:** Multivariate logistic regression analysis of microbial colonization of TCC.

**Variable**	**Model 1**		**Model 21** ^*^		**Model 3** ^**#**^	
	**OR**	***P***	**OR**	***P***	**OR**	***P***
Age	1.30 (0.58–2.92)	0.522	1.29 (0.57–2.89)	0.544	1.55 (0.63–3.80)	0.340
Sex	1.32 (0.57–3.05)	0.516	1.36 (0.58–3.19)	0.476	1.44 (0.59–3.53)	0.427
Diabetes mellitus	1.62 (0.70–3.74)	0.261	1.58 (0.68–3.68)	0.286	1.72 (0.68–4.35)	0.250
History of CRI	2.09 (0.83–5.23)	0.116	2.13 (0.85–5.35)	0.108	1.89 (0.71–5.08)	0.205
Catheter indwelling time	1.63 (0.72–3.67)	0.241	1.65 (0.73–3.73)	0.231	1.19 (0.49–2.91)	0.697
Fibrin sheath	2.22 (0.96–5.14)	0.062	2.31 (0.98–5.43)	0.056	1.99 (0.76–5.21)	0.162
WBC count (10^9^/l)	2.47 (1.10–5.58)	0.029	2.42 (1.07–5.47)	0.034	2.59 (1.05–6.42)	0.039
Central venous disease	3.37 (1.47–7.71)	0.004	3.06 (1.20–7.78) 4.13 (1.21–14.05)	0.019^a^ 0.023^b^	1.76 (0.44–7.02) 5.13 (1.41–18.63) 5.77 (1.04–32.04)	0.425^aa^ 0.013^bb^ 0.045^cc^

*The three models were adjusted for age, sex, diabetes mellitus, catheter indwelling time, fibrin sheath, history of CRI, and WBC count*.

* *Model 2 central venous disease is stratified into CVS and CVT*.

a *P = 0.019 for CVS*.

b *P = 0.023 for CVT*.

# *Model 3 CVS is graded into mild, moderate, and severe stenosis according to the degree of stenosis*.

aa *P = 0.425 for mild CVS*,

bb *P = 0.013 for mild CVS*,

cc *P = 0.045 for CVS*.

## Discussion

It is widely accepted that arteriovenous access is preferential to CVCs in most incident and prevalent hemodialysis patients. Several clinical practice guidelines on vascular access, including the Kidney Disease Outcome Quality Initiative (K/DOQI), and expert consensus indicate that catheters should be used in certain clinical circumstances, such as failure to establish arteriovenous access, patient preference considered valid after proper assessment, and some specific medical conditions (4, 18). According to the Chinese Expert Consensus on Hemodialysis Vascular Access (second edition), the proportion of catheters should be limited to within 10% (19). Current evidence suggests that catheters are associated with lower patency and higher complications than other vascular access types (20). CRI, for instance, is a well-known reason for all-cause mortality, hospitalization, and catheter dysfunction (21, 22). A number of recent publications reported that hemodialysis CVCs are associated with a two- to five-fold higher risk of infection-related hospitalization and a heavier economic burden due to CRBSI (23, 24). Understanding the pathogenesis of CRI can help us optimize catheter use. During catheter implantation, neglecting aseptic principles may result in microorganism migration. Relatedly, organisms transfer from the surrounding skin, health providers' hands, draping cloth next to the catheter exit site (known as an extraluminal pathway), or external or internal surfaces of catheter hubs or caps (extraluminal or intraluminal pathways) (17). Once organisms are attached to the catheter or accumulate in the fibrin sheath, biofilm production, a crucial and prerequisite step toward infection, is initiated. A mature biofilm is a unique self-sustaining colony of microorganisms protected by an exopolysaccharide matrix (25). The biofilm provides a suitable environment for the colonization and growth of organisms and helps them protect against antibiotics (17, 26, 27). In addition, a mature biofilm presents a hidden danger for subsequent disseminated infection and bloodstream infection. Studies have revealed a clear correlation between catheter microorganism colonization and CRBSI (12–14). The incidence of microorganism colonization in hemodialysis patients varies from 16.5 to 68% (12, 25, 28). From our observations, the rate of positive TCC tip cultures in patients without definite evidence of infection was 31.03%. The most common organisms (57.8%) detected were gram-positive bacteria, especially *S. epidermidis*.

A multivariate logistic regression analysis indicated that elevated WBC count, moderate to severe CVS, and CVT were independent risk factors for microbial colonization in catheters. CVS is often experienced by hemodialysis patients with arteriovenous access (29). In many cases, CVS is associated with indwelling catheters or other endovascular devices. The incidence of CVS varies greatly among different routes (30). The right internal jugular vein is preferable to the femoral vein for catheterization because of the lower risks for microbial colonization, CVS, and thrombotic complications (31). The incidence of CVS with a right internal jugular vein TCC was 26.2% in our study, which was relatively close to the 18–41% (29, 32, 33) reported in previous literature. Patients with CVS might have a higher chance of experiencing catheter dysfunction; therefore, more frequent nursing interventions were needed during the treatment and interval periods. This would undoubtedly increase the risk of microbes entering the catheter. Hemodynamic changes might be involved in the colonization process. Local lumen stenosis could result in greater blood flow resistance or even obstruction, which facilitates microorganism colonization in catheters. Furthermore, for reasons related or unrelated to CVS, hemodialysis patients with arteriovenous access often require percutaneous intracavitary treatment or surgical treatment to maintain access patency (34, 35). When a patient undergoes an operation or procedure, there is an additional opportunity for microorganisms to enter and spread, thereby amplifying the risk of microbial colonization in patients with stenotic lesions. In a retrospective study, 54 hemodialysis patients underwent venography at least half a year after catheter removal; the authors found that the incidence of CVS in patients with previous CRI was three-fold higher than that in the control group (75 vs. 28%) (36), which indicated that CVS and CRI interact and have a synergistic relationship.

CVT is also common in patients receiving hemodialysis through a CVC. The incidence of CVT was 11% in this study. However, Wilkin et al. (37) confirmed CVT by ultrasonography in 2003 and observed a higher incidence of 25.9% in 143 hemodialysis patients with a history of an indwelling TCC. Importantly, various factors, such as different assessment techniques, catheter tip positions, frequencies of catheterization, and lock solutions, contribute to the variations in CVT incidence. On the one hand, CVT promotes colonization by blocking blood flow and aggravating vortex flow; on the other hand, thrombi composed mainly of fibrin, fibronectin, collagen, laminin, and several types of immunoglobulin provide abundant nutrition and a suitable area for microbial colonization. Microorganisms represented by *S. aureus* and *S. epidermidis* are prone to adhering in this area (38). Moreover, coagulase released by these microorganisms activates the coagulation system and accelerates thrombosis, thereby promoting bacterial colonization and biofilm growth, ultimately resulting in the spread of bacteria in the blood (39).

The association between other factors (age, sex, diabetes mellitus, history of CRIs, and catheter indwelling time) and microbial colonization in the catheter was not demonstrated in our study. Among these characteristics, the effect of age on CRIs is controversial. Older age was considered a protective factor for CRBSI in the report of Murea et al. (40). In their opinion, the worse physical condition of elderly patients makes them less active than younger patients, placing less external mechanical stress on the catheter, which in turn maintains the integrity of the subcutaneous tunnel and reduces the introduction of skin microorganisms and the formation of catheter biofilms. Conversely, older age was a risk factor for microbial colonization and bloodstream infection in our previous study (41) because it led to more possibilities of malnutrition, impaired immunity, and frailty. Moreover, our present findings did not support a correlation between history of CRIs and microbial colonization of the catheter, which was similar to the finding of Delistefani et al. (42) that past infection was related to recurrent local catheter infection (exit or tunnel infection) but not to CRBSI.

## Conclusion

Our study indicates that an elevated WBC count and CVD were independent risk factors for TCC microbial colonization and may pave the way for large-scale, in-depth research on the association between CVD and CRI. Avoiding central venous catheterization and preventing catheter-related central venous lesions are crucial steps to reducing the incidence of microbial colonization in catheters.

## Data Availability Statement

The raw data supporting the conclusions of this article will be made available by the authors, without undue reservation.

## Ethics Statement

The studies involving human participants were reviewed and approved by the Institutional Review Board of the First Affiliated Hospital of Zhengzhou University. Written informed consent for participation was not required for this study in accordance with the national legislation and the institutional requirements.

## Author Contributions

XL devised the conceptual ideas and took the lead in writing the manuscript. XL, PW, and ZL contributed to the study design. YL, PZ, and PL collected the data from tunneled-cuffed catheter removal patients. XL and PW performed catheter removal and angiography diagnosis. YL, PW, and XL contributed to the discussion and interpretation of the results. YL, BC, and XL contributed to manuscript editing. All authors agreed that the entire concept and ownership of this work belong to PW. All authors approved the final manuscript.

## Conflict of Interest

The authors declare that the research was conducted in the absence of any commercial or financial relationships that could be construed as a potential conflict of interest.

## Publisher's Note

All claims expressed in this article are solely those of the authors and do not necessarily represent those of their affiliated organizations, or those of the publisher, the editors and the reviewers. Any product that may be evaluated in this article, or claim that may be made by its manufacturer, is not guaranteed or endorsed by the publisher.
